# Unusually young age distribution of primary hepatic leiomyosarcoma: case series and review of the adult literature

**DOI:** 10.1186/1477-7819-8-56

**Published:** 2010-07-01

**Authors:** Achraf Shamseddine, Walid Faraj, Deborah Mukherji, Nadim El Majzoub, Mohamed Khalife, Ayman Soubra, Ali Shamseddine

**Affiliations:** 1 Department of Surgery, HPB and Liver Transplantation Unit, American University of Beirut Medical Center, Beirut, Lebanon; 2 Department of Internal Medicine, Oncology Unit, American University of Beirut Medical Center, Beirut, Lebanon

## Abstract

**Background:**

Primary hepatic leiomyosarcoma is a rare disease diagnosed in older aged adults with a median age of 58 and occasionally in children with a history of immunosuppression.

**Methods:**

From 1998 to 2009, 215 patients were diagnosed with primary hepatic malignancies at our institution, 4 of which were diagnosed with primary hepatic sarcoma (1.8%). Three cases were primary hepatic leiomyosarcomas (LMS) and one case was primary undifferentiated embryonal sarcoma of the liver; median age 30 (range 20-39) years.

**Results:**

One patient is currently 12 months post-resection with no evidence of recurrence. Two patients passed away at 19 days and 22 months from small for size liver and tumor recurrence respectively.

**Conclusion:**

We have presented 3 cases of primary hepatic leiomyosarcoma diagnosed at our institution with an unusually young age distribution and no evidence of immunosuppression. These cases highlight the diagnostic and therapeutic challenges of this rare tumour.

## Introduction

Primary liver sarcoma is a rare tumor associated with rapid growth and poor prognosis. The majority of hepatic malignancies are carcinomas with sarcomas representing only 0.1% to 2% of primary hepatic cancers [1]. Presenting symptoms are nonspecific and the diagnosis is often made post-operatively or even post-mortem. Surgery for resectable tumors combined with adjuvant chemotherapy is the current standard of care however very little data exists outside case series due to the rarity of the disease.

Our institution is a leading tertiary referral centre for the treatment of hepatic malignancies in the region. We reviewed our cases of primary liver sarcoma and found three patients presenting with leiomyosarcomas (LMS) with an unusually young age distribution compared with previous reports of this disease. We present our cases and discuss the current literature including controversial management strategies such as liver transplantation.

## Patients

From 1998 to 2009, 215 patients were diagnosed with primary hepatic malignancies at our institution, 4 of which were diagnosed with primary hepatic sarcoma (1.8%). Three cases were primary hepatic leiomyosarcomas (LMS) and one case was primary undifferentiated embryonal sarcoma of the liver; with an age range of 20-39 years.

The first patient was a 25 year old female who presented with abdominal pain and a palpable right abdominal mass. A computed tomography (CT) scan revealed a 14 × 10 cm hepatic mass occupying segments 5,6,7,8 and compressing the right and middle hepatic veins. A right hepatectomy was subsequently performed. Histopathology revealed a primary hepatic leiomyosarcoma; immunohistochemistry showed positive staining for vimentin, desmin, smooth muscle actin (SMA) and muscle specific actin (MSA). The surgical margins were negative and the tumour was classified as stage I sarcoma under the American Joint Committee on Cancer (AJCC) classification system. The patient received adjuvant chemotherapy however the tumor recurred with distant metastasis and she passed away 22 months post resection.

The second patient was a 39 year old male who presented with abdominal pain associated with anorexia, fever and weight loss. A palpable abdominal mass was found on clinical examination. CT scan revealed a 27 × 22 × 17 cm mass in the right hepatic lobe. The lesion showed multiple internal hypodense areas compatible with necrosis. Fine needle aspiration was consistent with leiomyosarcoma. Immunohistochemistry showed positive staining for actin, desmin, and was strongly positive for vimentin. An extended right hepatectomy was performed and histopathological examination revealed a tumor size of 33 × 23 × 20 cm with positive surgical margins. One week post surgery, the patient developed fulminant liver failure secondary to small for size syndrome and passed away 19 days after surgery.

**Figure 1:**
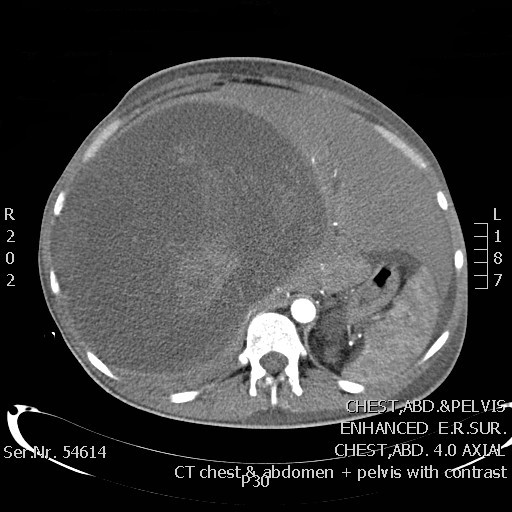
**CT scan of patient number 3 with primary hepatic sarcoma**.

The third patient was a 30-year old male who presented with abdominal pain associated with anorexia, weight loss, nausea and vomiting. A CT scan showed a large multi-lobulated lesion measuring 18 × 17.8 × 11.8 cm occupying almost the entire right hepatic lobe (figure 1). It appeared heterogeneous with cystic and soft tissue components. The initial diagnosis was of hydatid disease of the liver. A right hepatectomy was performed and histopathological examination defined the tumor as a stage IV LMS with metastasis to the abdominal wall. Immunohistochemistry showed diffusely positive staining for MSA, weakly positive for vimentin, and strongly and diffusely positive for SMA. Adjuvant chemotherapy was recommended and the patient continued his treatment at another institution. He received six cycles of chemotherapy and 30 sessions of radiotherapy. The patient is currently 12 months post-resection with no evidence of recurrence.

## Discussion

Leiomyosarcoma is a malignant mesenchymal tumour of myogenic origin rarely arising in the liver. Hepatic LMS has been reported to arise in children in association with acquired immunodeficiency syndrome (AIDS) and Epstein-Barr virus (EBV) [2,3]. We have reviewed the cases of hepatic LMS arising in adults reported in the English-language literature (Table 1) [4-14]. These show a median age of 58 with one case occurring in a patient immunosupressed following renal transplant and two cases occurring in patients previously treated for Hodgkin's lymphoma. The three cases of hepatic LMS diagnosed at out institution show an unusually young age distribution with a median age of 30 (range 25 to 30 years) and no history of immunosuppression or predisposing factors.

**Table 1:** Previous English-language reports of primary hepatic leiomyosarcoma in adults

Authors	Gender	Age	Past medical history	Follow-up
Liang 2009 [11]	F	44	Hepatitis B	Died 34 months
Giuliante 2009 [12]	M	26	Hodgkin's Lymphoma	Died 25 months
Matthei 2009 [1]	F	19		Died 73 months
	F	64		NED 181 months
	F	53		Died 21 months
	M	55		NED 133 months
	M	51		NED 144 months
	M	59		Died 45 months
	F	63		NED 133 months
Watanabe 2008 [13]	M	63		PMD
	F	49		PMD
Almogy 2004 [14]	F	58		Died 4 months
	F	63		Died 12 months
Fujita 2002 [15]	F	33	Prior renal transplant	NED 24 months
Iordanidis 2002 [16]	M	35		Died 3 months
Tsuji 2002 [17]	M	68	Hepatitis C	PMD
Soyer 1996 [7]	F	67		
Sato 2000 [18]	F	62		PMD
Holloway 1996 [19]	M	63		
Ferrozi 1996 [4]	M	50		
	F	35	Hodgkin's Lymphoma	NED 6 months
Baur 1993 [20]	F	69		Recurrence 10 years post-resection
Paraskevopoulos 1991 [21]	M	62		NED 5 months

NED: No evidence of disease, PMD: Post mortem diagnosis

Matthaei et al reported that survival in most patients with primary sarcoma of the liver is poor. However a long-term survival for more than 10 years after curative surgery (R0 resection) is possible [1]. One of our patients died soon after resection as a result of liver failure, one patient died 22 months after resection with metastatic disease and one patient remains free of disease 12 moths post-resection.

Diagnosis of hepatic LMS is challenging due to the non-specific nature of symptoms and lack of serological markers. Pre-operative histological diagnosis of liver tumors, particularly hepatocellular carcinomas is controversial due to the risk of needle-track seeding. Only one of our three patients had a pre-operative diagnosis of LMS made by FNA. Hepatocellular carcinoma (HCC) is the commonest primary live tumour and many patients who are diagnosed with hepatic sarcoma are initially treated for presumed HCC. In one of our cases the initial diagnosis was of hydatid disease of the liver which is the commonest cause of cystic liver lesions in this age group in our region.

The characteristics of hepatic LMS on CT or magnetic resonance imaging (MRI) are non-specific. CT imaging will invariably show a well-defined hypodense mass with peripheral enhancement with evidence of central necrosis or in some cases a cystic appearance. MRI may show homogeneous hypointensity on T-1 weighted images and heterogeneous hyperintensity on T-2 weighted images [15].

Histological examination of LMS reveals a tumour composed of intersecting bundles of spindle-shaped cells. Immunohistochemistry will show positivity for desmin, vimentin and smooth muscle actin but negative for keratin, S-100 protein and neuron-specific enolase.

The standard treatment for liver sarcomas is surgical resection followed by adjuvant chemotherapy however due to the rarity of the disease, there is very little data regarding optimal management and this remains empirical.

The efficacy of chemotherapy for LMS in general is unclear, particularly since older series include chemo-resistant gastrointestinal stromal tumors (GISTs) that are now treated with the c-kit inhibitor imatinib. The cytotoxic agents doxorubicin and ifosfamide are commonly used for many subtypes of soft tissue sarcoma both in the adjuvant and palliative setting. In a retrospective study of first-line chemotherapy for unresectable or metastatic LMS from different primary sites the overall response rate was 18%; median progression-free survival and overall survival were 3.8 months and 9.7 months respectively [16]. There is evidence that the combination of gemcitabine and docetaxel is active in uterine LMS with some evidence that it may be beneficial in the adjuvant setting [17]. The evidence of gemcitabine and docetaxel in advanced non-uterine LMS is less compelling and there are no studies evaluating adjuvant therapy non-uterine LMS.

Complete resection of the primary tumour (R0 status) is only potentially curative treatment for primary liver sarcoma. The largest case series of 22 primary hepatic sarcomas reported by Matthei et al found that no patient survived longer than 3 years after incomplete tumour resection [1].

Liver transplantation for primary hepatic sarcoma is controversial. Orthotopic liver transplantation (OLT) is now accepted as a valuable therapeutic option for early, unresectable HCC. Initial results showed high levels of tumour recurrence and disappointing short and long term survival rates however the seminal study by Mazzaferro et al in 1996 culminating in the development of the "Milan Criteria" established OLT as a viable treatment for HCC. These criteria stipulated that patients with a single tumour less than 5 cm or up to three tumors with the largest less than 3 cm with no evidence of vascular invasion, nodal or distant metastasis could be transplanted with acceptable rates of recurrence and overall survival comparable to OLT for benign disease. Transplantation for HCC within the Milan Criteria can achieve 4-year survival rates of 85% to 92% [18].

Liver transplantation for primary hepatic sarcoma has not been as successful as OLT for HCC. Due to concerns regarding needle-track seeding, most patients do not have a pre-operative histological diagnosis and the diagnosis of sarcoma is made on histological examination of the explanted liver. Husted et al reported the outcomes for 19 patients identified as undergoing OLT for primary or metastatic liver sarcoma. The 6 patients transplanted for primary hepatic sarcoma had primary hepatic angiosarcoma and had recurrence of disease at a median interval of 2 months (range 2 -10 months). Median survival was 5.7 months after transplantation (range 2.6-15.4 months) with all patients dying of disease recurrence. For the 13 patients receiving OLT for metastatic sarcoma to the liver, all patients had prior control of their primary malignancy before consideration of OLT and were free of extra-hepatic disease at the time of transplantation. Twelve patients had recurrence of disease after a median of 11.7 months (range 2-90 months), ten patients died of recurrent disease. The three long-term survivors have what is now termed GIST and are maintained on imatinib [19].

There are four reported cases of OLT for primary hepatic LMS. Saint-Paul et al. reported a case of primary hepatic LMS diagnosed post-transplant for presumed HCC who died 15 days post-operatively [20]. In the case series reported my Matthaei et al; two patients with hepatic LMS underwent OLT. A 19 year-old female with a 6 cm grade 2 hepatic LMS underwent OLT however developed diffuse pulmonary metastasis and died 73 months post-transplant. The second patient with hepatic LMS, a 52 year-old male, underwent OLT for two grade 2 lesions measuring 4 cm and 5 cm. He had local chest wall recurrence resected and was alive with no evidence of disease at 141 months [1]. The final reported case of OLT for hepatic LMS was a 44 year-old female with a presumed HCC on a background of hepatitis B measuring 5 cm. Histological examination of the explanted liver revealed hepatic LMS. Fourteen months post-transplant the patients developed recurrent disease metastatic to axillary lymph nodes. The immunosuppressive regimen was changed from tacrolimus to sirolimus and the patient survived for an additional 20 months before dying of extensive metastatic disease [21]. The interplay between immunosuppression and the development and progression of LMS is poorly understood however this case demonstrates the importance of immune status manipulation as a therapeutic strategy. Smooth muscle tumors are being increasingly recognized in immunodeficient children. One case EBV-related hepatic LMS arising post-liver transplant in a 2 year-old child responded to tapering of immunosuppression with the child remaining symptom-free with stable disease for 12 years [3].

## Conclusion

We have presented 3 cases of primary hepatic leiomyosarcoma diagnosed at our institution with an unusually young age distribution and no evidence of immunosuppression. These cases highlight the diagnostic and therapeutic challenges of this rare tumour. We have reviewed the literature and discussed treatment strategies including adjuvant chemotherapy and liver transplantation. Curative resection is the mainstay of treatment for hepatic leiomyosarcoma. In cases primary liver tumors with unusual features we would advocate pre-operative histological diagnosis in order to optimize management. Liver transplantation for hepatic LMS cannot be recommended due to high rates of tumour recurrence however in cases of LMS diagnosed on histopatholgical examination of the explanted liver or LMS arising post-transplant associated with immunosuppression, immune system modulation is an important strategy to consider. The role of adjuvant chemotherapy for hepatic LMS is unclear and remains empirical. In view of treatment advances in the treatment of uterine LMS, patients with non-uterine LMS should be offered treatment within clinical trials whenever possible to improve our understanding of this rare disease and improve outcomes for patients.

## Competing interests

The authors declare that they have no competing interests.

## Authors' contributions

AcS drafted the manuscript, WF and DM participated in the design of the study, NEM and AyS assisted with the collection of data and conceived of the study, MK and AlS participated in the design and coordination of the study. All authors read and approved the final manuscript.

## Consent

Written informed consent was obtained from the patient for publication of this case report and accompanying images. A copy of the written consent is available for review by the Editor-in-Chief of this journal.
